# Autopsy of a Boy Who Was Subject to Epileptic Fits, with Loss of Speech, Arising from Mechanical Injury of the Head

**Published:** 1845-05

**Authors:** 


### *Autopsy of a boy who was subject to Epileptic Fits, with loss of Speech, arising from mechanical injury of the head.*

Mr. Bunting's son, aged 8 years, has been subject to epilepsy since he was two years old. I have been in the habit of attending the patient during these attacks for about three years. In consequence of indisposition, I requested my young friend Dr. Snowden to visit him for me in his last sickness, and have been favoured with the following particulars of the autopsy, which was made by Dr. S. and will be read with interest.

Yours, &c.,

JNO. A. ELKINTON.

To the Editor of the Medical Examiner.

*Philadelphia, March 18th,* 1845.

DR. ELKINTON:

*Dear Sir:*—In compliance with your request I proceed to communicate, as correctly as circumstances will permit, the particulars of the case of which you were kind enough to desire me to make a post-mortem examination.

The history of the case previous to death, as obtained from the child's parents, is as follows. The child, a boy, when between one and two years of age, received (in consequence of a fall) an injury of the head, which was supposed to be a fracture. The situation and character of the osseous lesion I could not learn. The integuments were injured in the anterior portion of the left temporal region, immediately over the zygoma. Some weeks after the injury, a spiculum of bone, about three-fourths of an inch in length, and one or two lines in diameter, was expelled from the left ear. Previously to this time, and subsequently to the injury, there had been more or less sanguineous and purulent matter discharged from the ear. Subsequently to the injury, with gradually diminishing intervals, the boy was attacked with paroxysms of epileptic convulsions up to the time of his death, which occurred at about the age of eight years. Although of this age, he was entirely destitute of the faculty of speech. His powers of audition appeared to be unaffected.

At your request I saw the child, but did not prescribe, because it was very apparent that he was in articulo mortis at that time. He died about two hours after my visit.

Eighteen hours after death, assisted by Dr. McClain, I examined the brain and its coverings. Upon removing the integuments, no sign of previous lesion of the skull could be detected. In removing the superior portion of the skull considerable difficulty was experienced, owing to the unusually intimate adhesion existing between the dura mater and vitreous table in the temporal regions, and, particularly, in that of the left side. The dura mater in these regions presented evident traces of previous inflammation, and was considerably thickened, that on the left side being at least three times the ordinary thickness. The pia mater and the cerebral lamina of the arachnoid were also unusually dense in the same situation on the left side. The cerebral tissue appeared perfectly natural to the eye. On raising the brain we found evident marks of a previous fracture of the left lesser wing of the sphenoid bone, about three-fourths of an inch from its outer extremity. The course of the fracture appeared to have been directly in an antero-posterior direction, and a portion of the lesser wing had been completely isolated. The fragment had been reunited without material displacement. There had evidently been considerable inflammation at the seat of fracture. The dura mater at this point was very thick, and almost incorporated with the bone.

Upon examining the brain we found the vessels throughout its substance very much congested; and the lateral ventricles distended with serum. No unnatural appearance could be detected in any portion of the cerebral matter.

The conclusion in this case is, that the epileptic affection may have depended upon the unnatural thickness of the membranes covering the brain, or, perhaps, upon some organic alteration of the cerebral tissue itself. The former is, however, I believe, recognised by good authority as sufficient cause for the effects produced in this case.

The serum found in the ventricles deserves no attention, for it is probable it was effused at the time of death, or but a short time previously.

The absence of power to articulate words was probably dependant upon some organic alteration at the seat of origin of the motor nerves of the tongue, or, perhaps, in the course of the neves themselves.

Respectfully yours,

J. W. Snowden.

